# Treatment decision regret after precision prostatectomy: An analysis of patient‐reported outcomes predicting decision regret

**DOI:** 10.1002/bco2.476

**Published:** 2024-12-17

**Authors:** Kaushik P. Kolanukuduru, Wooju Jeong, Larisa Pistin, Firas Abdollah, Ash K. Tewari, Mani Menon

**Affiliations:** ^1^ Department of Urology Icahn School of Medicine at Mount Sinai New York New York USA; ^2^ Vattikuti Urology Institute Center for Outcomes Research, Analytics and Evaluation (VCORE) Henry Ford Hospital Detroit Michigan USA

**Keywords:** decision regret, patient‐reported outcomes, prostate cancer, quality of life, robotic surgical procedures

## Abstract

**Objectives:**

This study aimed to assess postoperative decision regret (DR) after precision prostatectomy (PP), a novel subtotal surgical technique for prostate cancer (PCa) that involves the preservation of the unilateral capsule and seminal vesicle, and to identify factors predictive of DR after PP.

**Materials and Methods:**

After a shared decision‐making process, 128 patients underwent PP for the treatment of localised PCa. Given the subtotal nature of the surgery, patients were informed about the possibility of a detectable prostate‐specific antigen and secondary treatment. Between 6 and 12 months of follow‐up, DR was analysed using the previously validated decision regret score (DRS). A univariable linear regression analysis was performed to analyse factors predictive of DR after PP.

**Results:**

Between 6 and 12 months after PP, objective measurements of DR were obtained on 64 patients who completed the DRS. At the time of DRS, 16 patients were impotent (SHIM < 17), while six were incontinent (≥1 pad/day). The median time to DRS was 10 months (IQR 7.5–11.8). Only two patients (3.1%) reported significant DR after PP (DRS > 25), while 53 patients (83%) reported no regret (DRS = 0). The median DRS was 0 (0–0). Incontinence and impotence at the time of DRS predicted higher DR after PP (incontinence estimate: 11.3 ± 3.2, *p* < 0.001; impotence estimate: 5.4 ± 2.3, *p* = 0.02).

**Conclusions:**

The incidence of DR after PP is low, with only 3% of patients reporting significant regret. Patients who are either incontinent or impotent after PP are more likely to regret their decision. Further studies with larger sizes and longer follow‐ups are required to measure the longitudinal trends in DR after PP.

## 1 INTRODUCTION

Robotic radical prostatectomy (RP) remains the preferred form of surgical treatment for prostate cancer (PCa) due to its excellent cancer control rates.^1^, ^2^ Unfortunately, RP results in long‐term erectile dysfunction (ED) and stress urinary incontinence (SUI) in 40%–60% and 5%–15% of men respectively.^3^, ^4^ After RP, patients report a decrease in quality of life, which is more pronounced in men who have higher levels of anxiety and depression before surgery.^5^ As a result, 20%–25% of patients regret their decision after choosing to undergo RP.^6^

Decision regret (DR) can be conceived as a negative emotion where patients wish they had made different decisions in the past, to attain a more favourable outcome in the present.^7^ Patients experiencing DR often attribute this emotion to an error in treatment selection, thus demonstrating that DR is intricately linked to the shared decision‐making process between the doctor and the patient.^8^ While the reporting of DR is disparate across the literature, most studies have identified ED and SUI to be major contributors to DR after the treatment of PCa.^9^, ^10^

Precision prostatectomy (PP), a novel surgical technique for PCa treatment, involves the preservation of the prostatic capsule and the seminal vesicle unilaterally to protect peri‐prostatic nerves involved in continence and erectile function.^11^ Studies exploring the functional outcomes after PP have shown that approximately 90% of patients regain potency after 1 year, with a secondary treatment rate of 9% at 36 months of follow‐up.^12^, ^13^, ^14^, ^15^, ^16^ The impact of improved functional outcomes on DR after PP remains unexplored. In this study, we sought to determine the incidence of DR after PP using an objective, validated tool and sought to determine factors that predict DR after PP.

## 2 PATIENTS AND METHODS

### 2.1 Study setting, design and participants

This study was conducted after institutional review board approval and included patients at two different institutions where the procedure was performed (HFH‐IRB#12507, HFH‐IRB#14531 and MSH‐IRB #24‐00275). PP was offered to patients who placed a high emphasis on maintaining erectile function and satisfied the following criteria: (1) unilateral dominant lesion of highest Gleason grade ≤ 4 + 3; (2) prostate‐specific antigen (PSA) ≤ 20 ng/mL; (3) clinical T stage ≤ T2; (4) no contraindications to anaesthesia; (5) life expectancy ≥ 10 years. Additionally, patients were required to undergo transperineal biopsies of the prostatic capsule using the KOELIS Trinity platform (Grenoble, France) to rule out the presence of cancer in the anticipated remnant (this protocol was implemented from the 52nd patient). These inclusion criteria were chosen as they were the criteria for focal therapy (FT) at the time of the study initiation. No exclusions were made based on prostate size or tumour location. In patients with Gleason 4 + 3 = 7 cancer (14%), conventional nerve sparing was performed on the diseased part of the prostate, while the rest received veil nerve sparing. PP was performed on the side opposite the dominant nodule.

### 2.2 Preoperative counselling

Patients were advised about the experimental nature of the procedure. They were informed that the risks of the procedure could not be accurately ascertained due to a lack of historical data. Patients were given reading materials about PP and other forms of treatment (RP, radiation [RT] and FT) to aid in making a well‐informed decision. Those who expressed interest in other forms of treatment were directed to the respective provider. All patients met with the surgeon at least twice before making a final decision. Patients were informed that while the postoperative functional results are anticipated to be favourable, the oncological outcomes are yet to be ascertained. The possibility of detectable PSA values after surgery (due to the subtotal nature of the procedure) and additional postoperative testing was discussed in detail. Preoperative counselling was centred around a shared decision‐making process between the surgeon and the patient to ensure that patients made a well‐informed decision that aligned with their values. Patients were encouraged to ask questions about the procedure and the postoperative course to ensure appropriate expectations after treatment. Following this, patients were advised to voluntarily reach out to the office after 1 week if they wanted to seek PP as their preferred form of treatment. All patients had to sign two consent forms before the procedure. The first consent reiterated details regarding the surgical technique, outcomes and the anticipated postoperative course. This was required at least 1 week before the day of surgery. The second was surgical consent on the day of surgery to reconfirm their understanding of the procedure and the associated risks.

### 2.3 Postoperative assessments and follow‐up

Following PP, patients returned to the office every 3–4 months for a PSA test and assessment of functional status. Erectile function was measured using the Sexual Health Inventory for Men (SHIM), while continence was measured using the Expanded Prostate Cancer Index Composite for Clinical Practice (EPIC‐CP) questionnaire (particularly Q3). Potency was defined as SHIM ≥ 17, while continence was defined as 0 pads/day.

Due to the subtotal nature of PP, we anticipated patients would have detectable PSA values after surgery. Since the PSA limits after PP are yet to be defined, all patients who met the AUA post‐RP criterion^17^ or the Huber post‐FT criterion^18^ for biochemical failure (BCF) after surgery underwent biopsies of the remnant prostatic tissue to rule out the presence of residual cancer.^19^ The presence of clinically significant PCa (csPCa) prompted secondary treatment (either completion prostatectomy or salvage RT/androgen deprivation therapy [ADT]). Patients with a detectable PSA and no evidence of cancer were followed with serial PSAs.

### 2.4 Covariates

Baseline patient and tumour characteristics included age, body mass index (BMI), race, Charlson comorbidity index score (CCI), education status, employment status, marital status, family history of PCa, family history of any cancer, preoperative PSA, SHIM, IPSS, biopsy Gleason score and clinical T stage. Postoperative variables recorded included decision regret score (DRS), months to DRS, postoperative SHIM, pad usage and secondary treatment rate.

### 2.5 Measurement of DR

DR was measured using the previously validated DRS.^20^ Between 6 and 12 months after surgery, all patients who underwent PP were contacted (via telephone and email) and offered the DRS questionnaire to obtain an objective measurement of DR as per the pre‐defined planned study protocol. Patients who did not respond or were unwilling to complete the questionnaire were excluded from the analysis. Patients were informed that the questionnaire pertained to their perioperative course alone. Once DRS was obtained, the scores were translated onto a scale of 0 to 100 to measure DR. DRS was measured as a continuous variable for the purposes of the statistical analysis. Using an alternate definition in line with previous studies that used the DRS in the context of PCa treatment, significant regret was defined as DRS >25 points.^6^, ^21^

### 2.6 Statistical analysis

Continuous variables were reported as medians with interquartile ranges, while categorical variables were presented as absolute numbers with proportions. A univariable linear regression analysis was performed to analyse factors associated with DR after PP. A two‐sided *p*‐value of ≤0.05 was considered statistically significant. The analysis was performed using the R programming software version 4.3.2.

## 3 RESULTS

### 3.1 Baseline characteristics

**FIGURE 1:**
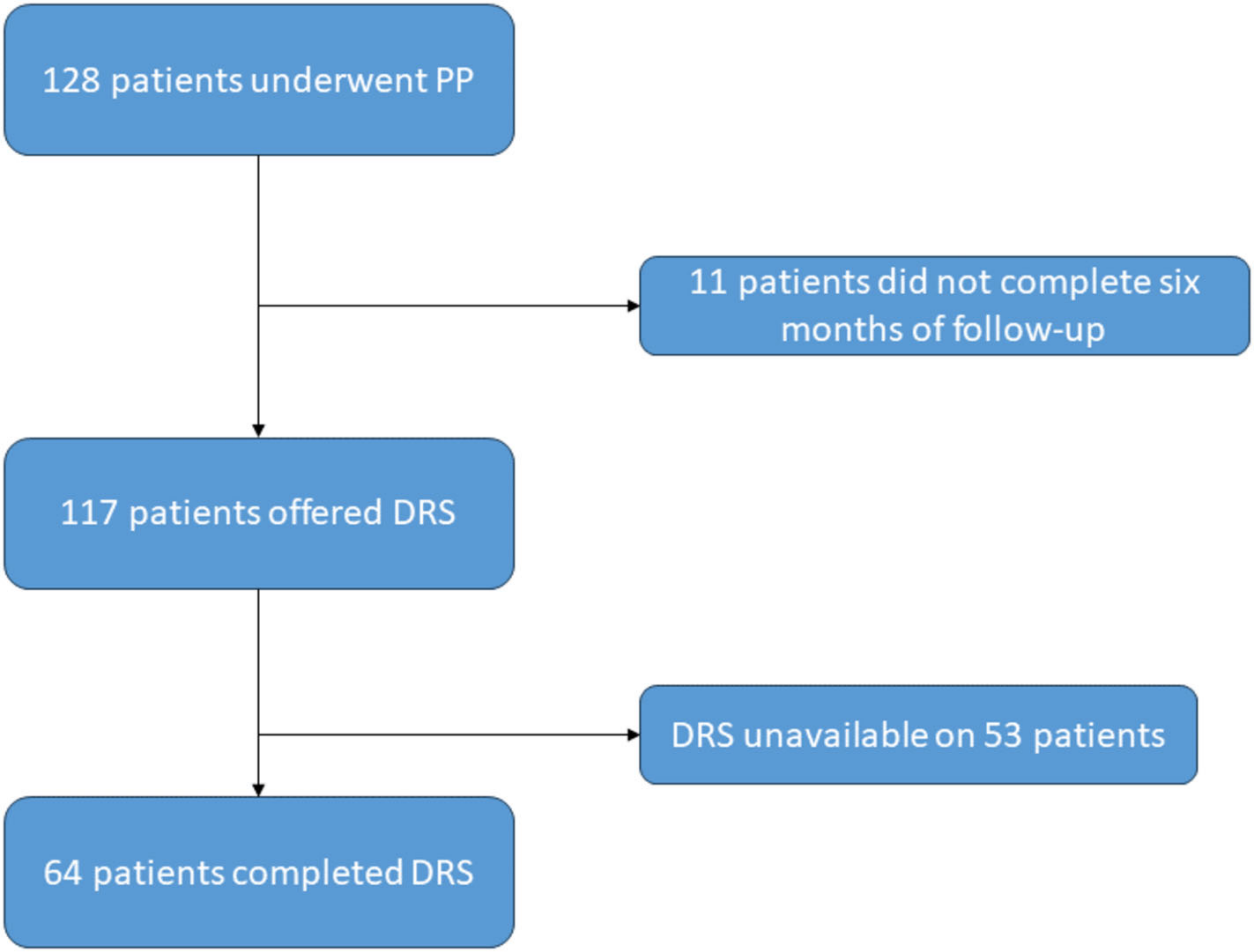
Study flow diagram. DRS, decision regret scale; RP, radical prostatectomy; PP, precision prostatectomy.

A total of 128 patients chose to undergo PP after a shared decision‐making process. Of these, 11 patients had not yet reached a follow‐up duration of 6 months. Of the remaining 117 patients, DRS was unavailable on 53 patients. Sixty‐four patients with DRS were included in the study (Figure 1). The comparative baseline characteristics of patients who completed the DRS and patients who did not have DRS are presented in Table S1.

The median age of patients included in the study was 60 years (IQR 54–64), and 53% of patients were actively employed at the time of treatment. Patients (75%) were married, and 37 (58%) had a family history of cancer, including 18 patients with a family history of PCa.

All patients were preoperatively continent; 91% were preoperatively potent. The median preoperative SHIM, IPSS composite score and IPSS QoL score were 22 (IQR 20–25), 4 (IQR 2–10) and 1 (IQR 0–2) respectively. The median preoperative PSA was 5.6 ng/mL (IQR 4.1–7.2). Seventy‐seven percent had Gleason 7 disease, and 78% had T1c cancer (Table 1). Of the 15 patients with Gleason 3 + 3 = 6 disease, 12 patients were not candidates for active surveillance according to the Epstein criteria^22^ and thus underwent treatment; the remaining three patients voluntarily chose PP as they were eager to have their cancer treated and did not want to be on surveillance.

**TABLE 1:** Baseline clinicopathological characteristics of patients undergoing precision prostatectomy (*n* = 64).

Parameter	Result
Age, median (IQR)	60 (54–64)
Body mass index, median (IQR)	27 (25–29)
Race, *n* (%)	
Caucasian	50 (78%)
African American	10 (16%)
Other	4 (6%)
Charlson Comorbidity Index Score, *n* (%)	
0–1	30 (47%)
2	22 (34%)
≥3	12 (19%)
Employment status, *n* (%)	
Employed	34 (53%)
Unemployed	6 (9.5%)
Retired	24 (37.5%)
Marital status, *n* (%)	
Married	48 (75%)
Divorced	8 (12.5%)
Single	8 (12.5%)
Family history of any cancer, *n* (%)	37 (58%)
Family history of prostate cancer, *n* (%)	18 (28%)
Preoperative PSA, median (IQR)	5.6 (4.1–7.2)
Biopsy Gleason Score, *n* (%)	
3 + 3	15 (23%)
3 + 4	40 (63%)
4 + 3	9 (14%)
Clinical T stage, *n* (%)	
T1c	50 (78%)
T2a	13 (20.5%)
≥T2b	1 (1.5%)
Baseline SHIM, median (IQR)	22 (20–25)
Baseline IPSS Composite Score, median (IQR)	4 (2–10)
Baseline IPSS QoL Score, median (IQR)	1 (0–2)
Preoperatively potent, *n* (%)*	58 (91%)
Preoperatively continent, *n* (%)**	64 (100%)

Abbreviations: IPSS, International Prostate Symptom Score; PSA, prostate‐specific antigen; QoL, quality of life; SHIM, Sexual Health Inventory for Men.

* ‐Potency was defined as a SHIM ≥ 17.

** ‐Continence was defined as the use of 0 pads/day.

### 3.2 Functional and oncological outcomes

At a median follow‐up of 64 months, 11% required secondary treatment, among whom five patients underwent completion prostatectomy and two patients underwent salvage RT + ADT [Table 2]. At the time of DRS, 16 patients (25%) were impotent, six patients (9%) were incontinent and 38 patients had a detectable PSA due to the subtotal nature of the procedure (with a down‐trending pattern).

**TABLE 2:** Postoperative functional, oncological and decision regret outcomes of patients after precision prostatectomy (*n* = 64).

Parameter	Result
Follow‐up duration (months), median (IQR)	64 (50.5–75.3)
Any secondary treatment after surgery, *n* (%)	7 (11%)
Impotent at the time of DRS, *n* (%)*	16 (25%)
Incontinent at the time of DRS, *n* (%)**	6 (9%)
Detectable PSA at the time of DRS, *n* (%)	38 (59%)
Significant decision regret (>25 points)	2 (3%)
DRS, median (IQR)	0 (0–0)
Months to DRS, median (IQR)	10 (7.5–11.8)

Abbreviations: DRS, decision regret score; PSA, prostate‐specific antigen.

* Potency was defined as a SHIM ≥ 17.

** Continence was defined as the use of no pads for urinary leakage.

### 3.3 DR outcomes and factors associated with DR

**FIGURE 2:**
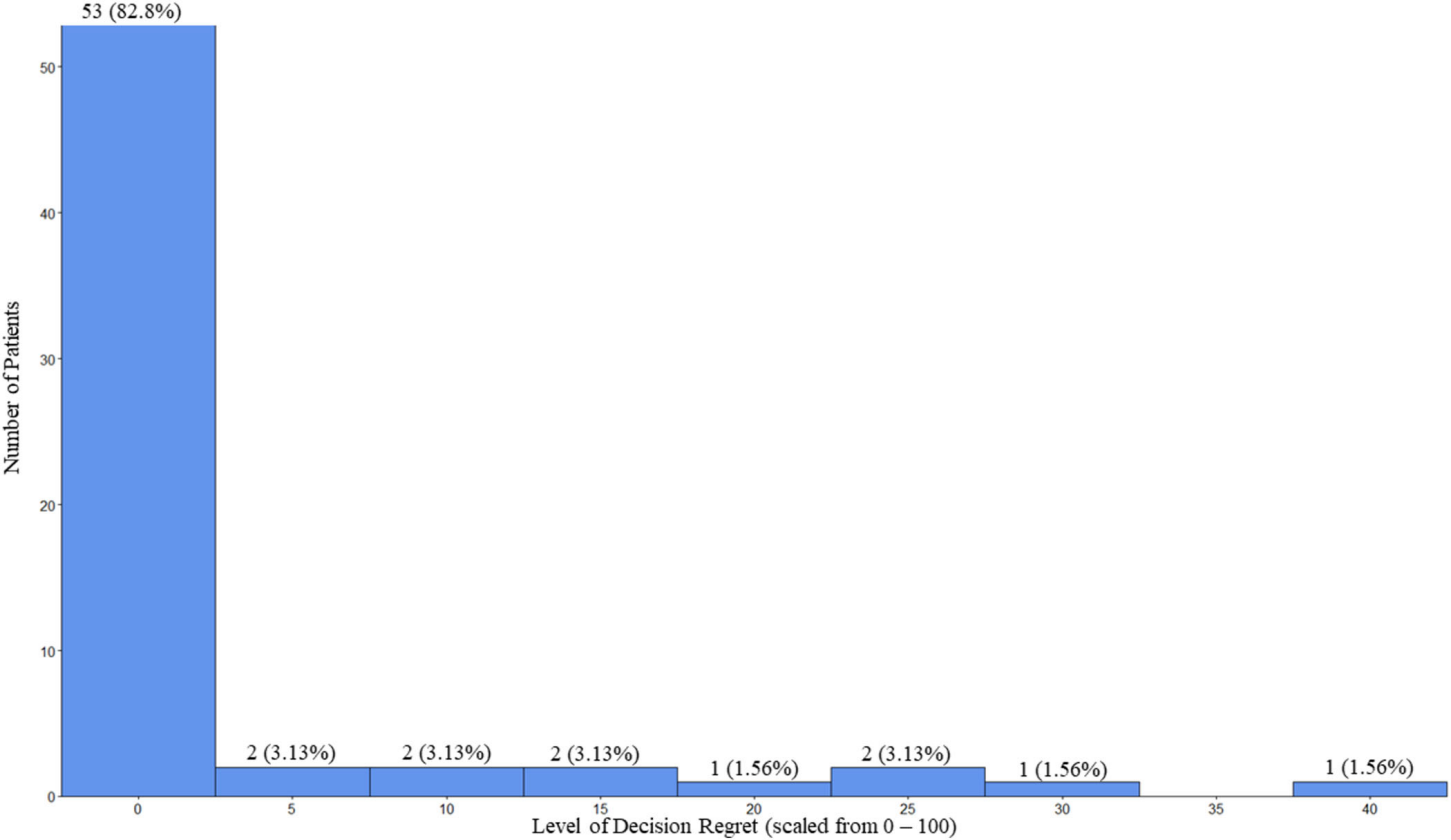
Histogram showing the absolute and relative proportions of decision regret (DR) after precision prostatectomy. DR has been measured using the decision regret scale, and scores have been translated onto a scale of 0–100 (a higher score means higher regret).

The median time to DRS was 10 months (IQR 7.5–11.8). Two patients (3%) reported significant DR after PP, while 53 patients (83%) reported a DRS = 0 (Figure 2). The median DRS for patients after PP was 0 (IQR 0–0). The DR outcomes of patients after PP in comparison to prior studies that used the DRS after cancer treatment are presented in Table S2.^6^, ^10^, ^21^, ^23^, ^24^, ^25^

Univariable linear regression analysis revealed that impotence at the time of DRS (estimate: 5.4 ± 2.3; *p* = 0.02) and incontinence at the time of DRS (estimate: 11.3 ± 3.2; *p* = 0.0009) were associated with higher DR (Table 3).

**TABLE 3:** Univariable linear regression examining predictors of decision regret after precision prostatectomy. The estimate along with the standard deviation and the corresponding *p*‐value are depicted.

Parameter	Univariable analysis	
	Estimate	*p*‐value
Age	−0.02 ± 0.16	0.9
Baseline SHIM	−0.04 ± 0.28	0.86
Baseline IPSS composite	−0.25 ± 0.13	0.06
Baseline IPSS QoL	1.25 ± 0.71	0.08
Months to DRS	−0.3 ± 0.35	0.42
Family history of any cancer	0.6 ± 2.1	0.77
Family history of PCa	−0.86 ± 2.2	0.7
Detectable PSA at the time of DRS	1.9 ± 2	0.37
Impotence at the time of DRS	5.4 ± 2.3	0.02
Incontinence at the time of DRS	11.3 ± 3.2	0.0009
Positive surgical margins	1.2 ± 2.1	0.56

Abbreviations: DRS, decision regret score; IPSS, International Prostate Symptom Score; PSA, prostate‐specific antigen; QoL, quality of life; SHIM, Sexual Health Inventory for Men.

## 4 DISCUSSION

The current investigation is the first study to analyse postoperative DR in patients undergoing PP. Our findings show that only 3% of patients report significant DR 1 year after PP, with a median DRS of 0 (IQR 0–0). Incontinence and impotence were associated with higher DR. Interestingly, detectable PSA at the time of DRS, family history of PCa and positive surgical margins did not predict higher regret.

Similar to our findings, studies that have measured DR after RP have identified SUI and ED to be among the primary determinants of DR. Lindsay et al. reported that 30% of patients experience DR after RP and that DR was associated with lower sexual and erectile function scores.^10^ Schroeck et al. found that DR was associated with lower urinary and hormonal domain scores; these differences are more pronounced for patients undergoing open RP when compared to RP.^26^ The lower incidence of DR in our study may be attributed to the superior functional outcomes after PP when compared to RP. The preservation of the additional peri‐prostatic nerves that are vital to continence and erectile function results in better functional outcomes, and thus lower DR after PP.

Patients tend to associate treatment success with PSA levels after treatment. Lunger et al. showed that BCF predicts DR in long‐term survivors after RP.^27^ Meissner et al. found that both early and late BCF were associated with higher DR after RP.^28^ Even in the context of FT, higher PSA after treatment is shown to be associated with higher DR.^21^ In our study, we found that detectable PSA after PP was not associated with higher DR. While larger studies with more numbers are required to make a definitive conclusion, our findings may be attributed to the shared decision‐making process and thorough preoperative counselling, where patients were clearly informed about the possibility of a detectable PSA after surgery and the need to undergo further testing. By informing patients clearly about the postoperative course, we adjusted their postoperative expectations, possibly resulting in lower DR.

Setting patient expectations is one of the most important components of preoperative counselling. Patients who discuss treatment options and are better informed about the potential side effects of treatment are noted to have lower levels of anxiety and DR after treatment.^29^ Retrospectively, patients with DR identify inadequate counselling to be the main cause of DR after RP.^30^ It is of utmost importance to properly counsel patients about their postoperative course and provide them with data regarding the outcomes and morbidities related to the treatment of PCa so that patients can make decisions that align with their values. In addition to the possibility of a detectable PSA after PP, patients were counselled extensively about the postoperative continence and erectile function outcomes. The expectations of the incontinent and impotent patients had not been met, resulting in higher DR in these patients. This suggests that patient satisfaction after PP is intricately linked to continence and erectile function after surgery, and patients at risk for incontinence and impotence must be followed carefully, with early intensified rehabilitation regimens if necessary.

This study is not without limitations. First, the DRS was unavailable in 53 patients; this may have introduced significant selection bias into our study. Second, we did not measure preoperative PSA anxiety or HADS scores and were thus not able to comment on the association between anxiety, depression and DR in this group of patients. Third, we had to limit our analysis to a univariable model to avoid overfitting due to the low incidence of DR in the study group. Finally, the DRS was administered between 6 and 12 months after surgery. Most patients who required secondary treatment underwent completion prostatectomy or radiation after 12 months, and thus, this study cannot comment on the effect of secondary treatment on DR in these patients. The findings of this study represent short‐term DR after PP. These limitations notwithstanding, our findings are both novel and informative and shed light on a critical aspect of the follow‐up of patients undergoing PP for the treatment of localised PCa.

## 5 CONCLUSIONS

Most patients after PP do not report DR, which may be attributed to the preservation of functional status after surgery. Only 3% of patients report significant DR after PP. DR is intricately linked to continence and potency after PP, and patients at risk for incontinence and impotence must be followed carefully with intensified rehabilitation protocols to mitigate DR. Future studies with larger numbers and longer follow‐up durations are required to truly measure longitudinal trends in DR after PP.

## AUTHOR CONTRIBUTIONS

KP Kolanukuduru: data curation, methodology, formal analysis, writing—original draft, final approval of manuscript. W Jeong: methodology, final approval of manuscript. L Pistin: data curation. F Abdollah: data curation, final approval of manuscript. A Tewari: methodology, final approval of manuscript. M Menon: project development, supervision, manuscript—review and editing, final approval of manuscript.

## CONFLICT OF INTEREST STATEMENT

AKT has served as a site‐PI on pharma/industry sponsored clinical trials from Kite Pharma Inc., Lumicell Inc., Dendron Pharmaceuticals LLC, Oncovir Inc., Blue Earth Diagnostics Ltd., RhoVac ApS., Bayer HealthCare Pharmaceuticals Inc. and Janssen Research and Development LLC. AKT has served as an unpaid consultant to Roivant Biosciences and advisor to Proxamo. He owns equity in Promaxo.

## Supporting information


**Table S1:** Comparative baseline characteristics of patients who completed the decision regret score (DRS) and patients who did not have DRS.
**Table S2:** Oncological studies examining the incidence of decision regret after cancer treatment using the decision regret score (DRS).

## Data Availability

Data and materials are not publicly available but are available by the corresponding author upon reasonable request.
