# Supplementary material for: Treatment decision regret after precision prostatectomy: An analysis of patient‐reported outcomes predicting decision regret
Source: BJUI Compass. 2024 Dec 17;6(1):e476. doi: 10.1002/bco2.476 (PMC11771488; doi:10.1002/bco2.476)
Supplement: Supplementary file 1 — Table S1: Comparative baseline characteristics of patients who completed the decision regret score (DRS) and patients who did not have DRS. Table S2: Oncological studies examining the incidence of decision regret after cancer treatment using the decision regret score (DRS). [file BCO2-6-e476-s001.docx]

**Supplementary Table 1:** **Comparative baseline characteristics of patients who completed the decision regret score (DRS) and patients who did not have DRS**

| **Parameter** | **DRS completed (n = 64)** | **DRS unavailable (n = 53)** | **p-value** |
| --- | --- | --- | --- |
| Age (years), median (IQR) | 59.5 (54 – 64.2) | 61.5 (55.8 - 66) | 0.46 |
| Body Mass Index, median (IQR) | 27 (25 - 29) | 28 (25.9 - 32) | 0.04 |
| Race, n (%):  White  African-American  Others | 50 (78.1%)  10 (15.6%)  4 (6.3%) | 36 (67.9%)  13 (24.5%)  15 (28.3%) | 0.01 |
| Charlson Comorbidity Index Score, n (%):  0 – 1  2  ≥ 3 | 30 (46.9%)  22 (34.4%)  12 (18.7%) | 24 (45.3%)  20 (37.7%)  9 (17%) | 0.5 |
| Preoperative PSA (ng/ml), median (IQR) | 5.6 (4.1 – 7.22) | 6.05 (4.26 – 8.3) | 0.29 |
| Biopsy Gleason Score, n (%):  3+3  3+4  4+3 | 15 (23.4%)  40 (62.5%)  9 (14.1%) | 10 (18.8%)  34 (64.2%)  9 (17%) | 0.68 |
| Clinical T Stage, n (%):  T1c  T2a  ≥ T2b | 50 (78.1%)  13 (20.3%)  1 (1.6%) | 40 (75.5%)  11 (20.8%)  2 (3.7%) | 0.37 |
| Baseline SHIM, median (IQR) | 22 (20 - 25) | 22 (19 - 25) | 0.75 |
| Baseline IPSS Composite Score, median (IQR) | 4 (2 - 10) | 5.5 (2 - 10) | 0.9 |
| Baseline IPSS QoL Score, median (IQR) | 1 (0 - 2) | 1 (0 - 2) | 0.44 |
| Preoperatively potent, n (%) * | 58 (90.6%) | 52 (98.1%) | 0.2 |
| Preoperatively continent, n (%) ** | 64 (100%) | 53 (100%) | 1 |

PSA – Prostate Specific Antigen, SHIM – Sexual Health Inventory for Men, IPSS – International Prostate Symptom Score, QoL – Quality of Life

*-potency was defined as a SHIM ≥ 17

**-continence was defined as the use of no pads for urinary leakage

**Supplementary Table 2:** **Oncological studies examining the incidence of decision regret after cancer treatment using the decision regret score (DRS)**

| **Study** | **Type of cancer** | **Number of patients** | **Treatment** | **Definition of Significant Regret** | **Incidence** |
| --- | --- | --- | --- | --- | --- |
| Van Stam et al.^6^ | Prostate | 195  98  141 | Surgery  Radiation  Active Surveillance | DRS > 25 points | 23%  37%  20% |
| Lindsay et al.^10^ | Prostate | 207 | Surgery | DRS ≥ 25 points | 23.6% |
| Ghoreifi et al.^21^ | Prostate | 143 | Focal Therapy | DRS > 25 points | 19.6% |
| Köksal et al.^23^ | Breast | 172 | Adjuvant Radiotherapy | DRS > 25 points | 13.9% |
| Sullivan et al.^24^ | Lung | 83  128 | Surgery  Radiation Therapy | DRS ≥ 25 points | 13%  36% |
| Nallani et al.^25^ | Head and Neck | 140 | Surgery, Radiation, Chemotherapy | DRS > 25 points | 29.3% |
| Kolanukuduru et al. | Prostate | 64 | Precision Prostatectomy | DRS > 25 points | 3.1% |
